# Application of diffusion tensor imaging in the diagnosis of post-stroke aphasia: a meta-analysis and systematic review

**DOI:** 10.3389/fpsyg.2023.1140588

**Published:** 2023-09-18

**Authors:** Weiming Zhu, Shizhe Deng, Hailun Jiang, Jieying Zhang, Boxuan Li, Wei Liu, Qingqing Jia, Wei Liu, Zhihong Meng

**Affiliations:** ^1^Clinical Department of Acupuncture, First Teaching Hospital of Tianjin University of Traditional Chinese Medicine, Tianjin, China; ^2^National Clinical Research Center for Chinese Medicine Acupuncture and Moxibustion, Tianjin, China; ^3^Department of Rehabilitation Medicine, The Second Affiliated Hospital of Shandong University of Traditional Chinese Medicine, Jinan, China; ^4^Department of Scientific Research, Shandong University of Traditional Chinese Medicine, Jinan, China

**Keywords:** diffusion tensor imaging, diagnosis, post-stroke aphasia, meta-analysis, system review, dual-stream language model

## Abstract

**Introduction:**

Diffusion Tensor Imaging (DTI) indicators of different white matter (WM) fibers and brain region lesions for post-stroke aphasia (PSA) are inconsistent in existing studies. Our study examines the consistency and differences between PSA tests performed with DTI. In addition, obtaining consistent and independent conclusions between studies was made possible by utilizing DTI in PSA assessment.

**Methods:**

In order to gather relevant studies using DTI for diagnosing PSA, we searched the Web of Science, PubMed, Embase, and CNKI databases. Based on the screening and evaluation of the included studies, the meta-analysis was used to conduct a quantitative analysis. Narrative descriptions were provided for studies that met the inclusion criteria but lacked data.

**Results:**

First, we reported on the left hemisphere. The meta-analysis showed that fractional anisotropy (FA) of the arcuate fasciculus (AF) and superior longitudinal fasciculus (SLF), inferior frontal-occipital fasciculus (IFOF), inferior longitudinal fasciculus (ILF), and uncinate fasciculus (UF) were decreased in the PSA group in comparison with the healthy controls (*p* < 0.00001). However, in the comparison of axial diffusivity (AD), there was no statistically significant difference in white matter fiber tracts in the dual-stream language model of the PSA group. Elevated radial diffusivity (RD) was seen only in the IFOF and ILF (*P*_IFOF_ = 0.01; *P*_ILF_ = 0.05). In the classic Broca’s area, the FA of the PSA group was decreased (*p* < 0.00001) while the apparent diffusion coefficient was elevated (*p* = 0.03). Secondly, we evaluated the white matter fiber tracts in the dual-stream language model of the right hemisphere. The FA of the PSA group was decreased only in the IFOF (*p* = 0.001). AD was elevated in the AF and UF (*P*_AF_ < 0.00001; PUF = 0.009). RD was elevated in the AF and UF (*P*_AF_ = 0.01; *P*_UF_ = 0.003). The other fiber tracts did not undergo similar alterations.

**Conclusion:**

In conclusion, DTI is vital for diagnosing PSA because it detects WM changes effectively, but it still has some limitations. Due to a lack of relevant language scales and clinical manifestations, diagnosing and differentiating PSA independently remain challenging.

**Systematic review registration:**

https://www.crd.york.ac.uk/PROSPERO/display_record.php?RecordID=365897.

## Introduction

Stroke is the world’s most common neurological disease, as well as the second leading cause of disability and death (Saini et al., 2021). Post-stroke aphasia (PSA), one of the most disruptive post-stroke cognitive deficits, has a high prevalence (Vitti and Hillis, 2021). 21%–38% of patients suffering from acute stroke have aphasia (Wade et al., 1986; Wertz, 1996; Kauhanen et al., 2000; Pedersen et al., 2004; Berthier, 2005). Right-handed patients’ PSA lesions are more common in their left hemisphere (LH) and less in their right hemisphere (RH) (Wade et al., 1986; Berthier, 2005). In addition to its affecting comprehension of auditory information, PSA can also affect oral expression, reading, and writing. It is a syndrome caused by the damage of various structures in the language formation stage or the disorder of physiological processes in the brain (Ali et al., 2015). The stage of speech formation in the brain refers to that before speech is spoken or written, its formation and external sound stimulus are transmitted and analyzed into the form of speech code with language characteristics and input into the Wernicke area, which is connected with various central regions through a large number of neural pathways and further transmitted to the auditory contact area. Through comprehensive analysis, these symbols and codes are transmitted along the arcuate fasciculus (AF) to the Broca’s area and begin to produce language movement and physiological performance. The AF is the most important and stable pathway. The inferior frontal-occipital fasciculus (IFOF), inferior longitudinal fasciculus (ILF), superior longitudinal fasciculus (SLF), and uncinate fasciculus (UF) are regarded as prominent neural tracts of language (Geschwind, 1970; Catani et al., 2005; Catani and Mesulam, 2008; Saur et al., 2008; Axer et al., 2013). Classical language models focus on cortical language areas. They include Broca’s area and Wernicke’s area. However, they cannot explain language problems caused by lesions outside these areas (Chang et al., 2015). In recent years, increasing studies have also focused on the connective anatomy of language or specific fibrous pathways connecting to the cortical and subcortical networks that control language (Dronkers et al., 2007; Sathian and Crosson, 2015). In pathological conditions, local necrotic tissue not only leads to grey matter injury but also the disruption of the integrity of the local fiber tracts of WM, even their displacement or recombination. Meanwhile, this WM alteration is closely related to the symptoms and severity of aphasia (Yang et al., 2017). Multimodal MRI has been widely used in PSA clinical studies over the last few years (Pustina et al., 2017; Allendorfer et al., 2021; Cherney et al., 2021; Iorga et al., 2021; Xu X. et al., 2021; Xu S. et al., 2021; Bae et al., 2022). In PSA, functional magnetic resonance imaging (fMRI), DTI, and other neuroimaging techniques demonstrate the recombination of the language matrix at structural and functional levels (Crosson et al., 2007; Jiménez de la Peña et al., 2018; Wilson et al., 2019). Different tissues can be tested using DTI, as a tool for noninvasive measurement by the diffusive motion of water molecules *in vivo*, which provides information about fractional anisotropy (FA), axial diffusivity (AD), radial diffusivity (RD), mean diffusivity (MD), and apparent diffusion coefficient (ADC) (Basser et al., 1994; Lope-Piedrafita, 2018). To facilitate the understanding of the diffusion tensor, it can be regarded as an ellipsoid. The three eigen quantities in the diffusion tensor that are perpendicular to each other can be regarded as axes perpendicular to each other in the ellipsoid, that is, one major axis and two minor axes representing depth and width, respectively. AD stands for mean diffusivity along the long axis of the ellipsoid. RD represents the mean diffusivity along the 2 minor axes perpendicular to the major axis in the ellipsoid. MD represents the overall level of molecular diffusion and diffusion resistance, independent of the direction of diffusion. FA is the most commonly used DTI parameter, which can quantitatively analyze anisotropy and further assess the degree of structural damage. FA was changed between 0 and 1, FA was close to 1, which could be regarded as the ellipsoid was infinitely elongated, indicating that the diffusion motion of water molecules was more restricted in the direction of the vertical fiber bundle, thus reflecting the more complete structure of the nerve fiber bundle and the better signal transduction ability. FA approaches 0 and the visible ellipsoid is a sphere, indicating that the more unrestricted diffusion movement made by water molecules reflects the more severe damage of nerve fiber bundles and the worse signal transduction ability. Using FA to reflect the difference of different regions of white matter in patients with stroke, and combining with ADC to describe the diffusion capacity of molecules in the body, reflecting the diffusion ability of water molecules. The increase of FA and ADC indicates the enhancement of nerve fiber conduction ability and the enhancement of water molecule diffusion ability, respectively, and vice versa. DTI offers a methodological innovation and challenge for the study of PSA that can better reflect the integrity of damaged WM fiber tracts (Crosson et al., 2007; Lope-Piedrafita, 2018). The mechanisms associated with PSA, one of the most complex dysfunctions after stroke, are not yet fully understood. Moreover, the microstructural changes in WM and neurobiological mechanisms underlying PSA are poorly understood. Currently, in addition to AF injury, disruption of multiple connections in the dorsal as well as ventral streams may directly contribute to language impairment in the case of WM injury (Yang et al., 2017).

Early on, this dual-stream language architecture was gradually recognized as the main network of WM in the language pathway (Frey et al., 2008; Weiller et al., 2011; Axer et al., 2013). Among them, anatomically based the dorsal streams mainly refers to the AF and the SLF. Functionally, the dorsal streams are primarily responsible for the speech processing from acoustic stimulation to the generation of physiological information for speech movement. At present, the observation of WM damage in PSA by DTI has focused more on studies of the AF (Breier et al., 2011; Kim et al., 2011; Wang et al., 2020; Bae et al., 2022). The AF is the main pathway connecting Broca’s area to Wernicke’s area. Damage to the AF may lead to several types of language impairment, such as varying degrees of damage or even disruption of the AF observed in conductive aphasia, motor aphasia, and complete aphasia (Bernal and Ardila, 2009; Ryu and Park, 2020; Cho and Jang, 2021). As part of the dorsal streams, the SLF is responsible for speech processing (Abel et al., 2015). In addition to connecting most of the cortical areas of the lateral hemisphere, it regulates higher-order cognitive functions, such as orientation and spatial awareness (van Hees et al., 2014). The ventral streams mainly consist of the IFOF, ILF, and UF, binding fiber tracts located in the anterior insula, external capsule, or polar capsule regions. Functionally, the ventral streams focus on the processing from sound to semantics, giving more profound meaning in terms of language comprehension (Hickok and Poeppel, 2004). The IFOF is the most extended contact fiber tract in the brain and is located above the UF which connects the frontal lobe to the temporal, parietal, and occipital lobes (Catani et al., 2002). Because of its extensive pathway, the IFOF is considered a multifunctional fiber and occupies a major part of the ventral streams, participating in semantic processing (Menjot de Champfleur et al., 2013). The IFOF and IOF run through the temporal lobe and are connected medially, liaising with the frontal and occipital lobes. It has been suggested that due to the wide distribution of IFOF, alterations in the brain’s multifunctional aspects of language processing, such as language, visuospatial processing, reading, memory, and conceptualization, occur after injury (De Benedictis et al., 2021). The UF, part of the ventral streams, is involved in a common cortical projection with the ILF and is closely related to functional language processing. A decrease in the ADC of UF injury is closelassociated with the severity of PSA (Zavanone et al., 2018).

DTI measures and identifies structural features of the brain in HCs and the PSA group based on anatomy to accurately diagnose or characterize clinical conditions, and further measures the biology of structures sensitive to clinical abnormalities to make predictions about clinical outcomes. It enables a more detailed study of the brain mechanisms of PSA through effective technical means. The three most common analytical methodologies for DTI are voxel-based analysis (VBA), tract-based spatial statistics (TBSS), and automated fiber quantification (AFQ). Currently, the TBSS is more commonly used, which improves the sensitivity, objectivity, and interpretability of DTI studies in subjects and addresses some of the limitations of the VBA (Bach et al., 2014). However, DTI studies for post-stroke aphasia still differ in their technical application and measurement. Certainly, more refined analysis methods aim to make the measurement of WM integrity more accurate. Based on this, this paper presents meta-analysis and systematic review of studies related to DTI in PSA with the main fiber tracts in the dual-stream language model architecture, which we believe is meaningful. We will examine different indicators of fiber tracts and evaluate the correlation between the degree of integrity impairment of varying fiber tracts in PSA and language impairment from multiple perspectives to obtain a consensus and independent view between studies.

## Materials and methods

The study follows the PRISMA 2020 statement and is registered with PROSPERO (CRD42022365897) to improve transparency and quality.

### Article retrieval

**Figure 1:**
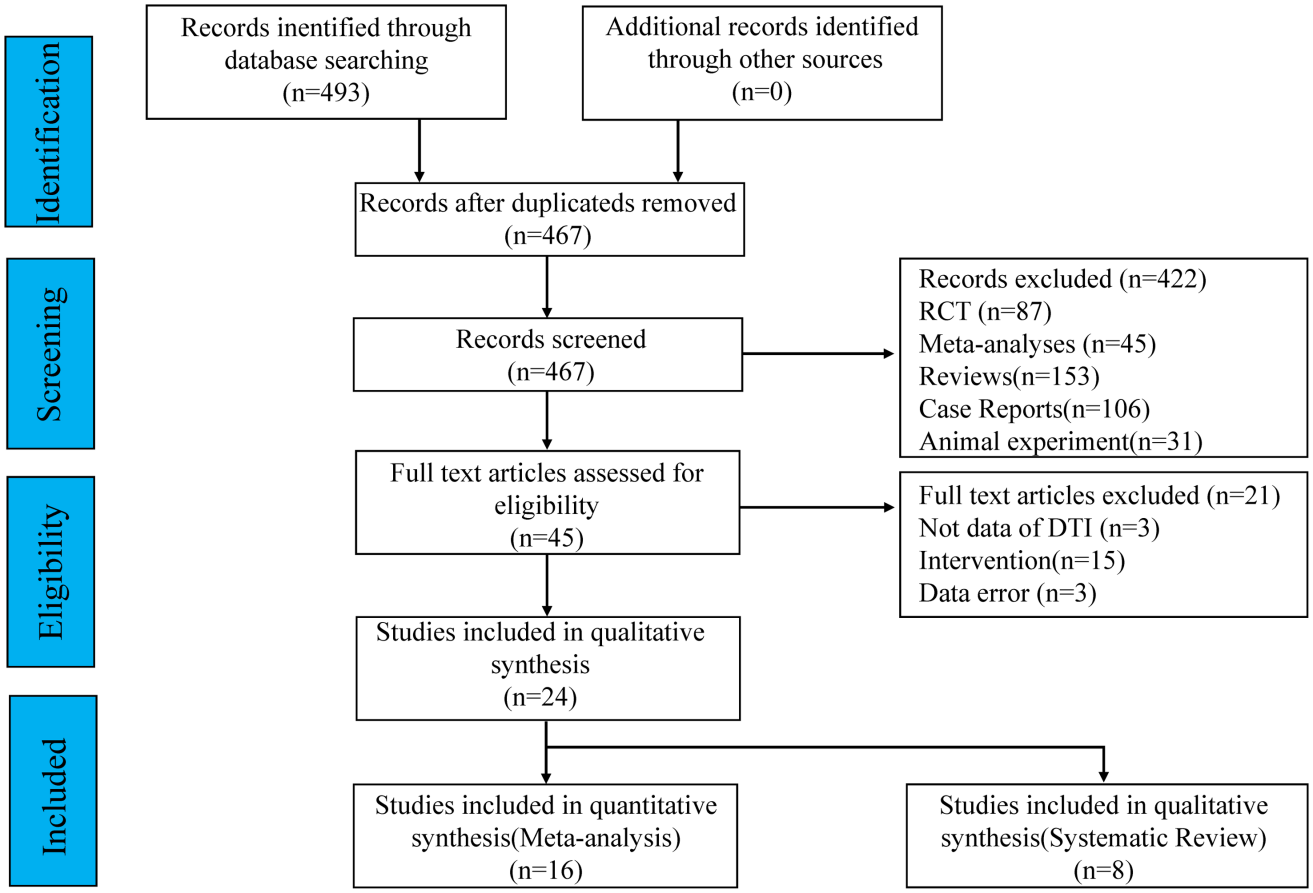
Flowchart of literature retrieval and screening.

The aim of this paper is to summarize the literature related to DTI in PSA research. We conducted advanced searches in the Web of Science database, PubMed database, and China National Knowledge Infrastructure (CNKI) database, by using the term of “[‘aphasia’ (title/abstract) or ‘post-stroke aphasia’ (title/abstract) or ‘word deafness’ (title/abstract) or ‘stroke’ (title/abstract) or ‘cerebrovascular accident’ (title/abstract)] and [‘diffusion tensor imaging’ (title/abstract) or ‘DTI’ (title/abstract) or ‘brain imaging’ (title/abstract) or ‘neuroimaging’ (title/abstract)].” The search criteria were from database creation to October 31, 2022. After reading the abstracts of 493 articles, an initial screening yielded 46 articles, and 447 articles were excluded for the following reasons: (1) the studies were animal experiments, such as studies in rats or monkeys; (2) the studies were case reports; (3) the studies were duplicate literature; (4) the studies were non-stroke-induced speech disorders, such as primary aphasia; (5) the studies were non-imaging studies. The complete process of searching and including studies is shown in Figure 1.

### Inclusion and exclusion criteria

Through initial screening, we searched and read 46 full-text articles in the literature. All of the included studies were case-control studies and utilized MRI for DTI sequence scans and description of the major language-related WM. All patients in the included studies had aphasia due to single or multiple LH strokes and were right-handed. By reading the full text, we again excluded 22 studies. The main exclusion criteria were as follows: (1) the participants were not healthy controls; (2) studies with incomplete data results or significantly incorrect data from DTI experiments; (3) studies for which statistical analysis of data was not possible; (4) studies using functional MRI and other sequences; (5) the participants were aphasia due to bilateral strokes; and (6) the participants were left-handedness. Among the 24 included studies, we performed a meta-analysis of 16 of them. The FA, AD, and RD of the AF, SLF, IFOF, ILF, and UF in the dual-stream language model were described in these 16 studies and are expressed as mean ± standard deviation. The remaining 8 studies were reviewed systematically because specific values were unavailable or were only reported for the whole brain or some brain regions. We report the basic information of these 24 studies, containing the gender distribution of participants, age, years of education, type of stroke, type of aphasia, language assessment scale, whether it was a first stroke and etc. (see Table 1).

**Table 1:** Basic characteristics of literature research.

Study	Sex (M/F)		Age		Type of stroke	Time from onset (days)	Type of aphasia	Educational level (years)		Language evaluation	First-ever stroke
	AG	CG	AG	CG				AG	CG		
Yang (2016)	6/12	8/12	53.67 ± 13.66	54.05 ± 8.43	I/H	9.7 ± 5.3	NR	8.67 ± 1.24	8.45 ± 1.47	ABC	Yes
Lee et al. (2018)	23/0	NR	69.3 ± 1.3	NR	NR	>180	NR	NR		WAB	Yes
Yao et al. (2020)	9/1	14/3	54.90 ± 12.58	54.00 ± 15.26	NR	428 ± 52	NR	11.40 ± 3.60	14.29 ± 4.96	WAB	Yes
Geva et al. (2015)	10/5	12/6	64 ± 11	64 ± 6	I (14)/H (1)	>180	NR	NR		CAT	NR
Wang et al. (2020)	13/2	6/3	52.73 ± 11.71	53.4 ± 9.3	NR	83.1 ± 37.8	NR	11.2 ± 4.07	10.44 ± 3.25	ABC	Yes
Ivanova et al. (2016)	18/19	5/6	54 ± 10.53	53 ± 8.56	NR	NR	NR	NR		ASA	NO
Zhang et al. (2018)	9/5	5/6	62	50	I (14)	13	GA	9	11	ABC	Yes
Koyama and Domen (2016)	8/2	8/13	NR	NR	I (10)	NR	BA (4); WA (5); AA (1)	NR		WAB	NR
Kim et al. (2011)	2/3	2/5	48.4	49.6	I (3)/H (2)	NR	BA	NR		WAB	Yes
Kourtidou et al. (2021)	19/6	19/5	56.4 ± 13.2	53.6 ± 11.5	NR	NR	NR	13.52 ± 3.6	14.4 ± 2.9	BDAE	Yes
Xing et al. (2017)	14/26	16/11	59.6 ± 10.1	59.8 ± 14.3	NR	45.3 ± 38.6	NR	16.3 ± 2.9	16.3 ± 2.6	WAB	NR
Wang et al. (2019)	3/15	3/9	52.5 ± 10.8	53.4 ± 9.3	I (11)/H (7)	NR	BA (13); GA (2); WA (3); AA (1)	NR		ABC	Yes
Chen et al. (2015)	6/5	10/6	53.4 ± 7.2	49.8 ± 5.3	I (11)	NR	BA	NR		ABC	Yes
Yuan (2017)	49/51	51/49	55.5 ± 1.1	57.3 ± 0.9	NR	NR	NR	NR		NR	NR
Lu et al. (2022)	8/12	8/12	67.52 ± 8.63	66.89 ± 8.49	I (20)	NR	CA	8.93 ± 3.09	9.12 ± 3.36	ABC	Yes
Wang et al. (2022)	5/13	NR	67.6 ± 10.2	NR	NR	NR	NR	NR		WAB	Yes
Zhuang et al. (2008)	11/19	9/8	67 ± 12	72 ± 7	I (30)	4 ± 2	BA	NR		NR	NR
Luo et al. (2021)	7/8	9/6	62.4	59.8	I (15)	NR	BA	NR		NR	Yes
Liu (2021)	0/10	0/10	55.7 ± 9.56	53.18 ± 9.13	I (10)	53.4 ± 21.06	NR	12.6 ± 2.6	14.4 ± 3.3	WAB	Yes
Li (2021)	8/33	10/20	59.41 ± 11.3	55.23 ± 9.21	NR	62.51 ± 41.71	NR	10.9 ± 4.04	12.37 ± 2.54	CRRCAE	Yes
Wang (2009)	12/24	12/17	64.6 ± 9.5	59.9 ± 10.8	I (36)	NR	BA	NR		ABC	NR
Chen (2021)	9/9	8/10	57.61 ± 10.52	57.06 ± 7.53	I (18)	NR	NR	8.67 ± 3.07	8.17 ± 2.07	WAB	Yes
Yang (2016)	6/11	8/12	53.53 ± 14.06	54.05 ± 8.43	NR	11.7	BA (2); CA (6); AA (9)	8.71 ± 1.26	8.45 ± 1.47	WAB	Yes
Zhang (2017)	4/7	5/6	57.9 ± 11.9	52.4 ± 18.1	I (11)	14 ± 2	GA (7); BA (1); WA (2); AA (1)	9.5 ± 4.0	10.9 ± 4.7	ABC	Yes

M, male; F, female; NR, not report; I, ischaemic; H, haemorrhagic; WAB, Western Aphasia Battery; ABC, Aphasia Battery of Chinese; CAT, Comprehensive Aphasia Test; BDAE, Boston Diagnostic Aphasia Examination; CRRCAE, China Rehabilitation Research Center Aphasia Examination; ASA, Assessment of Speech in Aphasia GA, Global aphasia; BA, Broca aphasia; WA, Wernicke aphasia; CA, conduction aphasia; AA, anomic aphasia.

### The meta-analysis and system review

As part of the quality assessment, we used the Newcastle–Ottawa scale (NOS), which is a tool to assess the study’s quality and includes eight indicators, such as participant selection, exposure and significant factors, and comparability controls (see Table 2). Two investigators independently evaluated the included literature for each of the 24 studies included in this meta-analysis and systematic review, and discussions and decisions were made by Professor ZM over any disagreements. A summary of the studies that were included in the meta-analysis, including the type of study, statistical analysis, number of participants, basic parameters of MRI, language assessment as well as region of interest (ROI), was provided (see Table 3). Since the ROI selected varies significantly from study to study, we selected several ROI with the great deal of commonality. As part of the dual-stream language model, we selected major fiber tracts such as AF/SLF in the dorsal stream and IFOF/ILF/UF in the ventral stream. We extracted the FA, AD, and RD of the above fiber tracts, respectively. Since motor aphasia was the majority of the included studies, we also selected FA and ADC for the Braco area. Some of the ROI were included in fewer studies but are still of interest. The data from the included literature studies were statistically analyzed using Rev. Man 5.3 software. It is appropriate to use the mean difference (MD) or standard mean difference (SMD) as the effect measure for continuous variables and use the risk ratio (RR) for Binary variables. Both situations give a 95% confidence interval (CI). For experiments with significant heterogeneity (*I*^2^ > 50%, *p* ≤ 0.1), the random-effect model was chosen instead of the fixed factor model. Researchers used sensitivity analysis or subgroup analysis to investigate sources of heterogeneity and funnel plots to detect bias in literature.

**Table 2:** The Newcastle–Ottawa scale (NOS) used to evaluate the quality of the included literature.

Study	Selection				Comparability	Exposure			Score
	Adequate definition of cases	Representativeness of the cases	Selection of controls	Definition of controls	Comparability of cases and controls on the basis of the design or analysis	Ascertainment of exposure	Same method of ascertainment for cases and controls	Non-response rate	
Yang (2016)	★	★	★	★	★★	☆	★	★	8
Lee et al. (2018)	★	★	☆	★	★★	☆	★	★	7
Yao et al. (2020)	★	★	☆	★	★★	☆	★	★	7
Geva et al. (2015)	★	★	☆	★	★★	☆	★	★	7
Wang et al. (2020)	★	★	☆	★	★★	☆	★	★	7
Ivanova et al. (2016)	★	★	☆	★	★★	☆	★	★	7
Zhang et al. (2018)	★	★	☆	★	★★	★	★	★	8
Kim et al. (2011)	★	★	☆	★	★★	☆	★	★	7
Koyama and Domen (2016)	☆	★	☆	★	★☆	☆	★	★	6
Kourtidou et al. (2021)	☆	★	☆	★	★★	☆	★	★	6
Xing et al. (2017)	★	★	☆	★	★☆	☆	★	★	6
Wang et al. (2019)	★	★	☆	★	★★	☆	★	★	7
Chen et al. (2015)	☆	★	★	★	★☆	☆	★	★	6
Yuan (2017)	☆	★	☆	★	★☆	☆	★	★	5
Lu et al. (2022)	☆	★	★	★	★★	☆	★	★	7
Wang et al. (2022)	★	★	☆	★	★★	☆	★	★	7
Zhuang et al. (2008)	☆	★	☆	★	★☆	☆	★	★	5
Luo et al. (2021)	☆	★	☆	★	★☆	☆	★	★	5
Liu (2021)	★	★	☆	★	★★	☆	★	★	7
Li (2021)	★	★	☆	★	★★	☆	★	★	7
Wang (2009)	★	★	☆	★	★☆	☆	★	★	6
Chen (2021)	★	★	☆	★	★★	☆	★	★	7
Yang (2016)	★	★	★	★	★★	☆	★	★	8
Zhang (2017)	★	★	☆	★	★☆	☆	★	★	6

The Newcastle–Ottawa scale (NOS) used to evaluate the quality of the included literature. According to the corresponding evaluation content, “★” is used to indicate that it conforms to the content of the entry, and “☆” is used to indicate that it does not meet the content of the entry or is not mentioned. Each “★” represents one point. The total score of the article is not <5 points to be qualified.

**Table 3:** Statistical table of studies included in the meta-analysis.

References	Type of study	Participant	Field strength	TE	TR	*b*-value	Analysis	Language evaluation	ROI
Lee et al. (2018)	Case control	23PSA + 10HC	3T	92	9700	1000	TBSS	+	SLF, ILF
Wang et al. (2020)	Case control	15PSA + 9HC	3T	68	5000	1000	ROI	+	AF, SLF
Ivanova et al. (2016)	Case control	37PSA + 11HC	1.5T	95	6000	1000	TBSS	+	AF, SLF, ILF, IFOF, UF, CST
Zhang et al. (2018)	Case control	14PSA + 11HC	3T	92	13000	1000	TBSS/AFQ	+	SLF, AF, IFOF, UF, ILF
Koyama and Domen (2016)	Case control	10PSA + 21HC	3T	83	7000	1000	TBSS	+	AF
Kourtidou et al. (2021)	Case control	25PSA + 24HC	3T	68	7299	1000	ROI	+	AF, SLF
Wang et al. (2019)	Case control	18PSA + 9HC	3T	68	5000	1000	ROI	+	AF, SLF
Chen et al. (2015)	Case control	18PSA + 9HC	3T	95	3600	1000	ROI	+	Broca
Yuan (2017)	Case control	100PSA + 100HC	—	—	—	—	ROI	—	Broca
Lu et al. (2022)	Case control	20PSA + 20HC	3T	83	8000	800	FT	+	AF, Broca, Wernicke
Wang et al. (2022)	Case control	18PSA + 18HC	3T	87	8500	1000	TBSS	+	SLF
Zhuang et al. (2008)	Case control	30PSA + 17HC	3T	68	8672	800	FT	+	Broca
Luo et al. (2021)	Case control	15PSA + 15HC	3T	75	12000	1000	FT	+	Broca, AF
Wang (2009)	Case control	36PSA + 29HC	3T	93	5700	800	FT	+	Broca, AF
Chen (2021)	Case control	38PSA + 18HC	3T	86	1700	1000	AFQ	+	AF, IFOF
Zhang (2017)	Case control	14PSA + 11HC	3T	—	13000	1000	TBSS	+	AF, SLF, ILF, IFOF, UF

ROI, regions of interest; TBSS, tract-based spatial statistics; AFQ, automated fiber quantification; FT, fiber tracking; AF, arcuate fasciculus; SLF, superior longitudinal fasciculus; ILF, inferior longitudinal fasciculus; IFOF, inferior frontal-occipital fasciculus; UF, uncinate fasciculus.

## Results

### Literature quality evaluation

First, since the types of our included studies were all case-control studies, we evaluated the quality of the included studies using NOS. Among the eight entries we listed, “★” was used to indicate that the entry content was met and “☆” indicated that the entry content was not met or not mentioned, respectively, according to the corresponding evaluation content. Each “★” represents one point. The standard for evaluating the quality of literature is 5 points or higher. Statistically, all 24 studies included in the systematic review and meta-analysis met the criteria (see Table 2).

### Basic characteristics of studies in the literature

Twenty four qualified studies (Zhuang et al., 2008; Wang, 2009; Kim et al., 2011; Chen et al., 2015; Geva et al., 2015; Ivanova et al., 2016; Koyama and Domen, 2016; Yang, 2016; Xing et al., 2017; Yang et al., 2017; Yuan, 2017; Zhang, 2017; Lee et al., 2018; Zhang et al., 2018; Wang et al., 2019, 2020, 2022; Yao et al., 2020; Chen, 2021; Kourtidou et al., 2021; Li, 2021; Liu, 2021; Luo et al., 2021; Lu et al., 2022) involving 557 patients with PSA and 504 healthy individuals were published. In addition to 11 studies published in English databases, 13 studies were published in Chinese databases. Each study had explicit inclusion and exclusion criteria. Of these, 21 studies specified language assessment for patients. Nine studies used the Western Aphasia Battery (WAB). Eight studies used the Aphasia Battery of Chinese (ABC). One study used the China Rehabilitation Research Center Aphasia Examination (CRRCAE). One study used the Comprehensive Aphasia Test (CAT). One study used the Assessment of Speech in Aphasia (ASA). One study used the Boston Diagnostic Aphasia Examination (BDAE). Eleven studies specified the type of PSA, including motor aphasia, sensory aphasia, conductive aphasia, and complete aphasia. They identified the type of aphasia in the enrolled patients on the basis of AQ values and clinical presentation. The evaluation method is to evaluate the aphasia type of patients by combining the scores of spontaneous speech, comprehension, repetition and naming. Among them, the type of aphasia in 5 studies was motor aphasia. The type of aphasia studied in one study was global aphasia. The type of aphasia studied in one study was conductive aphasia. Three studies included multiple types of aphasia. The other 13 studies did not make a clear distinction between types of aphasia. Therefore, one of the aims of this study is to explore the objective detection and evaluation methods to make up for the limitations of the validity and reliability of the scale evaluation. Diffusion tensor imaging may have certain advantages in this regard, but the design and methods of current research still need to be improved and optimized. Fourteen studies specified the nature of the stroke, including hemorrhagic and ischemic. Seventeen studies specified that the included patients had a first stroke. The basic characteristics of the 24 eligible studies are listed in Table 2.

## The meta-analysis

Sixteen studies reported FA, AD, and RD for major fiber tracts and ROI. Therefore, we performed a meta-analysis of these 16 studies and collected MRI data from these studies, as shown in Table 3. Twelve studies reported a field strength of 3 T. One study reported 1.5 T. Six studies reported TBSS as the DTI analysis method. Two studies reported AFQ as the DTI analysis method. Four studies reported the fiber tracking as the DTI analysis method. Five studies used computational methods that labeled ROI with fiber tracts or local areas of the brain.

### Dorsal streams

**Figure 2:**
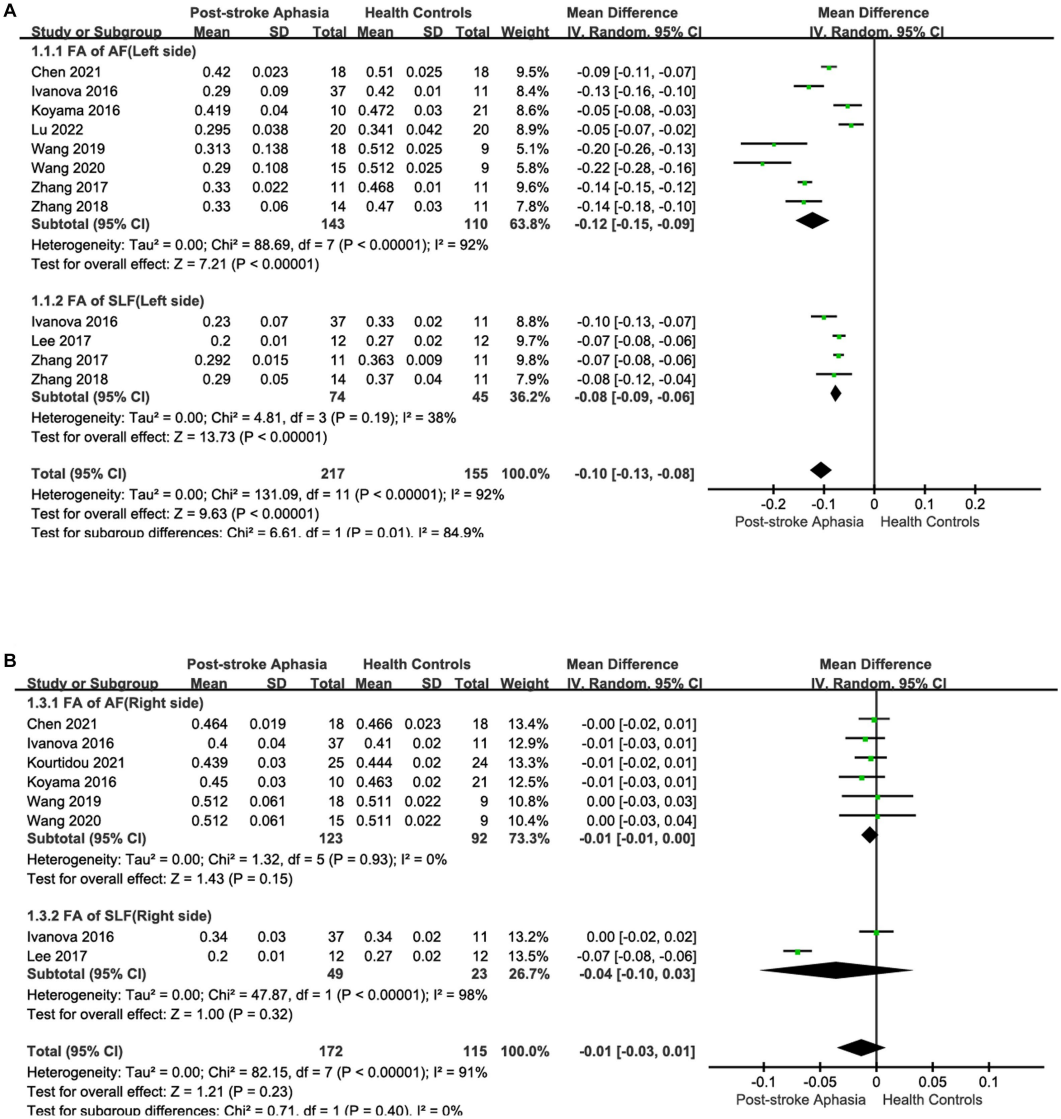

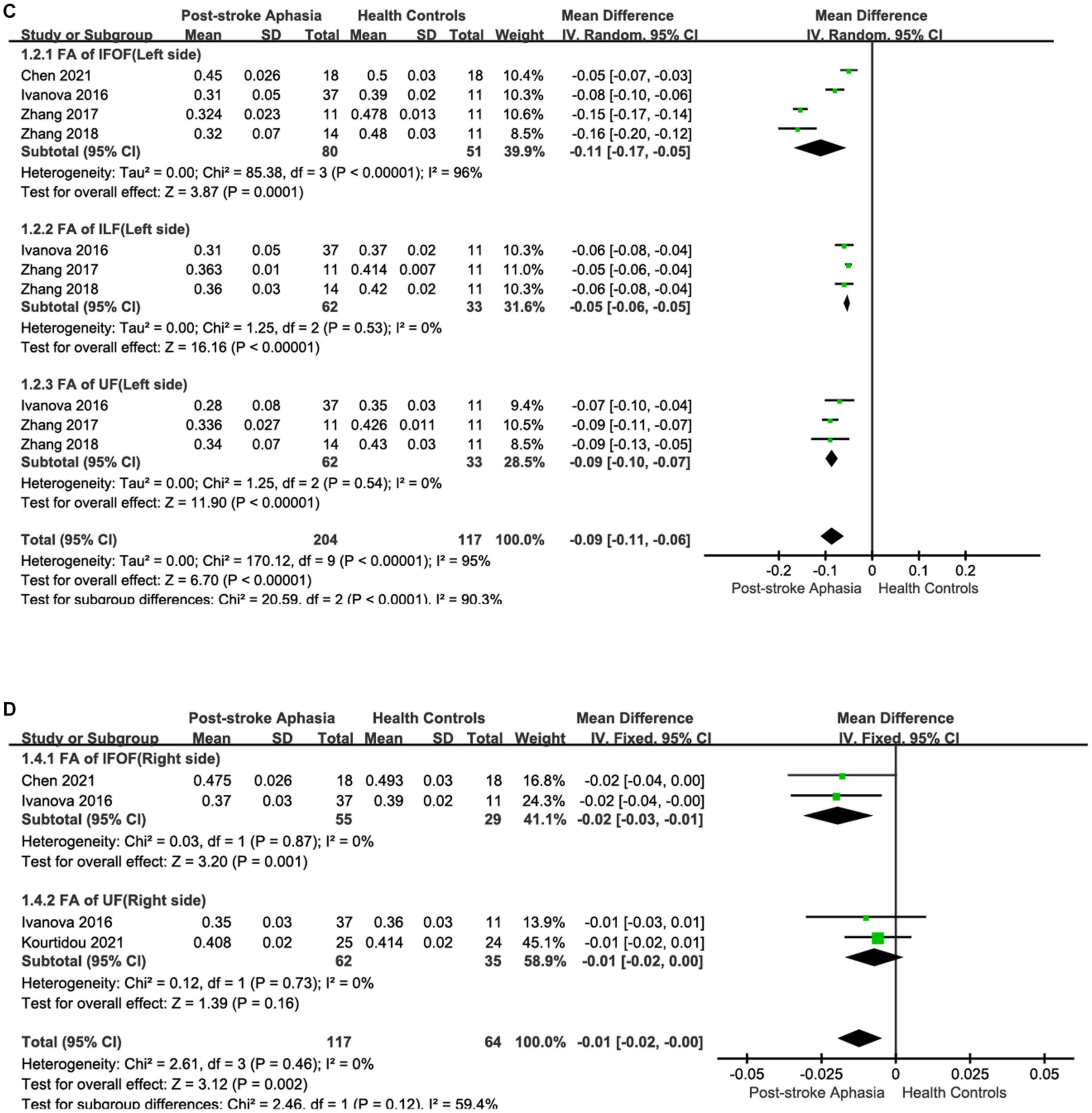
Subgroup analysis of the FA in the LH and RH. **(A)** Comparison of FA in the dorsal streams, including AF and SLF, in the LH of PSA and HCs. **(B)** FA of the dorsal streams, including AF and SLF, in the RH of PSA and HCs. **(C)** Comparison of FA in the ventral streams, including IFOF, ILF and UF in the LH of PSA and HCs. **(D)** Comparison of FA in the ventral streams, including IFOF and UF, in the RH of PSA and HCs.

**Figure 3:**
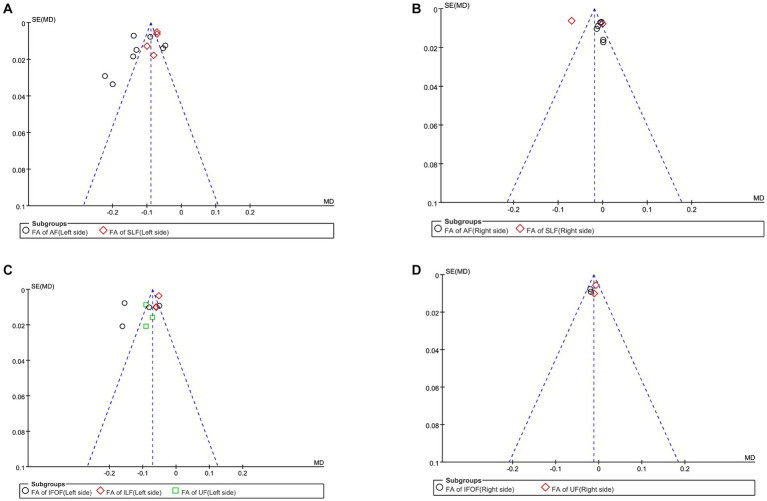
Funnel plot of the fractional anisotropy. **(A)** Funnel plot comparing the FA of the dorsal streams in the LH of PSA and HCs. **(B)** Funnel-plot comparing the FA of the dorsal streams in the RH of PSA and HCs. **(C)** Funnel plot comparing the FA of the ventral streams in the LH of PSA and HCs. **(D)** Funnel-plot comparison of the FA of the ventral streams in the RH of PSA and HCs.

First, we performed a the meta-analysis of the FA of AF and SLF in the dorsal streams. To compare the overall integrity of fiber tracts in the dorsal streams, we grouped AF and SLF in the LH into the same subgroup. It is noteworthy that our subgroups were based on the commonality of their locations, but there were significant differences between the studies within the groups on factors other than the subgroup factors. After subgroup analysis, we found significantly lower FA values for fiber tracts in the LH in the PSA group compared to HCs (MD = −0.10; 95% CI = −0.13 to −0.08; *p* < 0.00001; *I*^2^ = 92%; 372 participants), but heterogeneity was high (see Figure 2A). It appears that the funnel plots between the studies are asymmetrical, indicating publication bias (see Figure 3A). Eight studies reported FA values for AF in the LH. Based on a random-effects model, the FA was significantly lower in the PSA group than in the HC group (MD = −0.12; 95% CI = −0.15 to −0.09; *p* < 0.00001; *I*^2^ = 92%; 253 participants), but heterogeneity was high (see Figure 2A). Four studies reported FA for SLF in the LH. Based on a random-effects model, PSA group had lower FA than HC group (MD = −0.08; 95% CI = −0.09 to −0.06; *p* < 0.00001; *I*^2^ = 38%; 119 participants) (see Figure 2A). We also performed a statistical analysis of FA for dorsal streams in the RH. The results showed reduced FA for fiber tracts in the RH compared to HCs (MD = −0.01; 95% CI = −0.03 to −0.01; *p* = 0.23; *I*^2^ = 91%; 372 participants), but the differences did not reach statistical significance. According to a meta-analysis of the six included studies, the PSA group had a trend toward lower FA for AF, but the differences did not reach statistical significance (MD = −0.01; 95% CI = −0.01 to 0.00; *p* = 0.15; *I*^2^ = 0%; 215 participants) (see Figure 2B). Similarly, we performed a statistical analysis of the FA for SLF in the RH. The results showed a trend toward lower FA in the PSA group (MD = −0.04; 95% CI = −0.10 to −0.03; *p* = 0.32; *I*^2^ = 98%; 72 participants), but the difference was not statistically significant (see Figure 2B). Funnel plots showed asymmetry prior to the study and were more pronounced relative to the LH, indicating greater publication bias (see Figure 3B).

**Figure 4:**
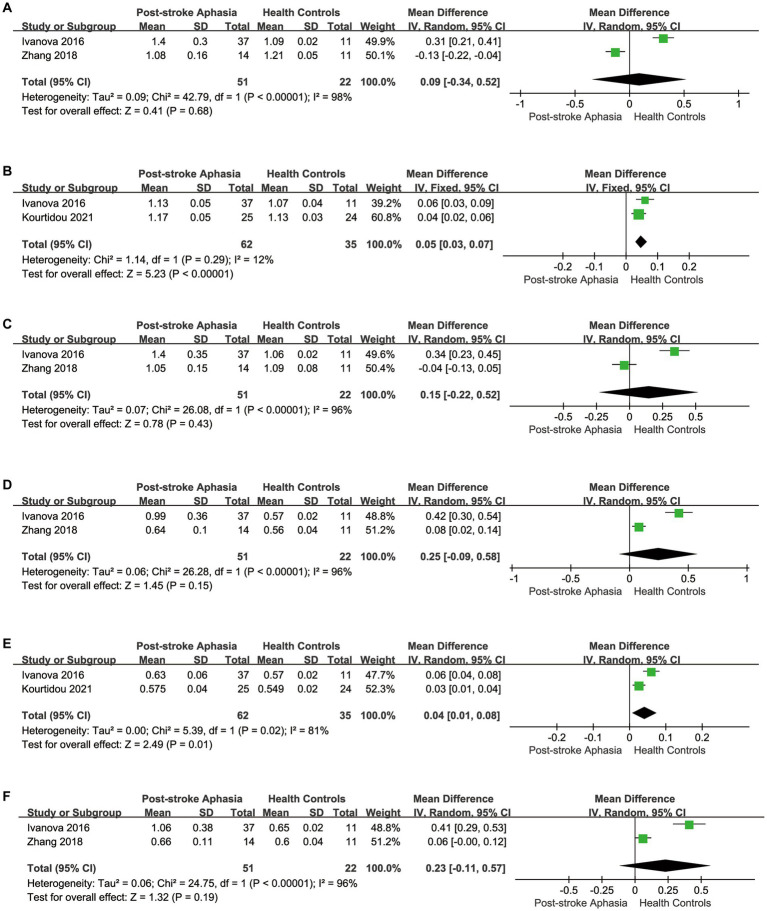
Meta-analysis of the AD and RD in the dorsal streams. **(A)** Comparison of AD for AF in the LH of PSA and HCs. **(B)** Comparison of AD for AF in the RH of PSA and HCs. **(C)** Comparison of AD for SLF in the LH of PSA and HCs. **(D)** Comparison of RD for AF in the LH of PSA and HCs. **(E)** Comparison of RD for AF in the RH of PSA and HCs. **(F)** Comparison of RD for SLF in the LH of PSA and HCs.

Second, we performed a the meta-analysis of the AD of AF and SLF in the dorsal streams. Two studies reported AD for AF in the LH. The meta-analysis was performed using a random-effects model, patients with PSA had higher AD values than HCs (MD = 0.09; 95% CI = −0.34 to 0.52; *p* = 0.68; *I*^2^ = 98%; 73 participants), but the difference was not statistically significant (see Figure 4A). Two studies reported AD for AF in the RH. According to a fixed-effects model for the meta-analysis, the PSA group had a higher AD compared to the HC group (MD = 0.05; 95% CI = 0.03 to 0.07; *p* < 0.00001; *I*^2^ = 12%; 97 participants) (see Figure 4B). Two studies reported AD for SLF in the LH. Based on a random-effects model for the meta-analysis, the PSA group had higher AD than HCs (MD = 0.15; 95% CI = −0.22 to 0.52; *p* = 0.43; *I*^2^ = 96%; 73 participants), but the difference was not statistically significant (see Figure 4C).

Finally, we conducted a meta-analysis of the RD of AF and SLF. Two studies reported the RD for AF in the LH. Based on the meta-analysis’ random-effects, the RD was higher in the PSA group than in HCs (MD = 0.25; 95% CI = −0.09 to 0.58; *p* = 0.15; *I*^2^ = 96%; 73 participants), but the differences did not reach statistical significance (see Figure 4D). Two studies reported the RD for AF in the RH. Based on the meta-analysis’ random-effects, the PSA group had higher RD than HCs (MD = 0.04; 95% CI = 0.01 to 0.08; *p* = 0.01; *I*^2^ = 81%; 97 participants), but heterogeneity was high (see Figure 4E). Two studies reported the RD for SLF in the LH. The meta-analysis used random effects to calculate the results, the PSA group had higher RD than HCs (MD = 0.23; 95% CI = −0.11 to 0.57; *p* = 0.19; *I*^2^ = 96%; 73 participants), but the differences did not reach statistical significance (see Figure 4F).

### Ventral streams

First, we conducted a meta-analysis of the FA of IFOF, ILF, and UF in the ventral streams. Consistent with the purpose of the previous study, we grouped IFOF, ILF, and UF in the LH into the same subgroup in order to compare the integrity of fiber tracts in the ventral streams. After subgroup analysis, it was shown that the FA of fiber tracts in the LH was reduced compared to HCs (MD = −0.09; 95% CI = −0.11 to −0.06; *p* < 0.00001; *I*^2^ = 95%; 321 participants), but the heterogeneity was high (see Figure 2C). Funnel plots showed asymmetry between studies and were more pronounced relative to dorsal streams, indicating a more pronounced publication bias (see Figure 3C). Four studies reported the FA for IFOF in the left hemisphere. The meta-analysis used random effects to calculate the results, the FA was significantly lower in the PSA group than in HCs (MD = −0.11; 95% CI = −0.12 to −0.10; *p* < 0.00001; *I*^2^ = 96%; 131 participants), but the heterogeneity was high (see Figure 2C). Three studies reported the FA for ILF in the LH. The meta-analysis used random effects to calculate the results, the FA was significantly lower in the PSA group than in HCs (MD = −0.05; 95% CI = −0.06 to −0.05; *p* < 0.00001; *I*^2^ = 0%; 95 participants) (see Figure 2C). Similarly, we performed a statistical analysis of the FA in the LH in UF in the 3 studies. The results showed that the FA was significantly lower in the PSA group than in HCs (MD = −0.09; 95% CI = −0.10 to −0.07; *p* < 0.00001; *I*^2^ = 0%; 95 participants) (see Figure 2C). The meta-analysis used fixed-effects to analyze the fiber tracts in the RH. The results showed reduced FA for fiber tracts in the RH in the PSA group relative to HCs (MD = −0.01; 95% CI = −0.02 to −0.00; *p* = 0.002; *I*^2^ = 0%; 181 participants) (see Figure 2D). Funnel plots showed asymmetry between studies, indicating publication bias (see Figure 3D). We also performed a statistical analysis of the FA for IFOF in the RH. A The meta-analysis of the 2 included studies using a fixed-effects model showed that the FA was lower in the PSA group than in HCs (MD = −0.02; 95% CI = −0.03 to −0.01; *p* = 0.001; *I*^2^ = 0%; 84 participants) (see Figure 2D). Two studies reported the FA for UF in the RH. The meta-analysis used fixed-effects to calculate the results, there was a trend toward lower FA in the PSA group (MD = −0.01; 95% CI = −0.02 to 0.00; *p* = 0.16; *I*^2^ = 0%; 95 participants), but the differences did not reach statistical significance (see Figure 2D).

**Figure 5:**
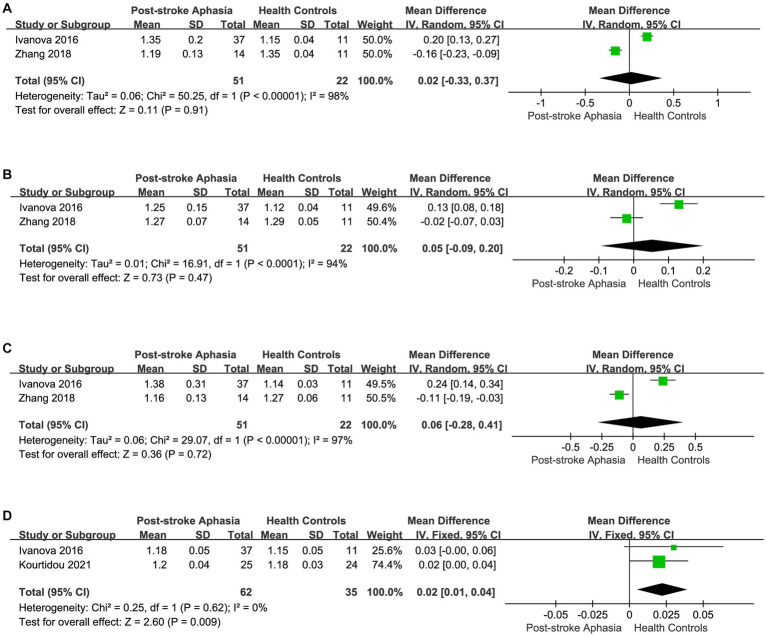
Meta-analysis of the AD in the ventral streams. **(A)** Comparison of AD for IFOF in the LH of PSA and HCs. **(B)** Comparison of AD for ILF in the LH of PSA and HCs. **(C)** Comparison of AD for UF in the LH of PSA and HCs. **(D)** Comparison of AD for UF in the RH of PSA and HCs.

Second, we performed a the meta-analysis of the AD of IFOF, ILF, and UF in the ventral streams. Two studies reported the AD for IFOF in the LH. Using a random-effects model for The meta-analysis, there was a trend toward higher AD in the PSA group (MD = 0.02; 95% CI = −0.33 to 0.37; *p* = 0.91; *I*^2^ = 98%; 73 participants), but there was no statistically significant difference (see Figure 5A). Two studies reported the AD values for ILF in the LH.The meta-analysis used random-effects to calculate the results, the PSA group had higher AD than HCs (MD = 0.05; 95% CI = −0.09 to 0.20; *p* = 0.47; *I*^2^ = 94%; 73 participants), but the difference was not statistically significant (see Figure 5B). Two studies reported the AD for UF in the LH. Using a fixed-effects model for The meta-analysis, there was a trend toward higher AD in the PSA group (MD = 0.06; 95% CI = −0.20 to 0.41; *p* = 0.72; *I*^2^ = 97%; 73 participants), but the differences did not reach statistical significance (see Figure 5C). Two studies reported the AD for UF in the RH. The meta-analysis used fixed-effects to calculate the results, the PSA group had higher AD than HCs (MD = 0.02; 95% CI = 0.01 to 0.04; *p* = 0.009; *I*^2^ = 0%; 97 participants) (see Figure 5D).

**Figure 6:**
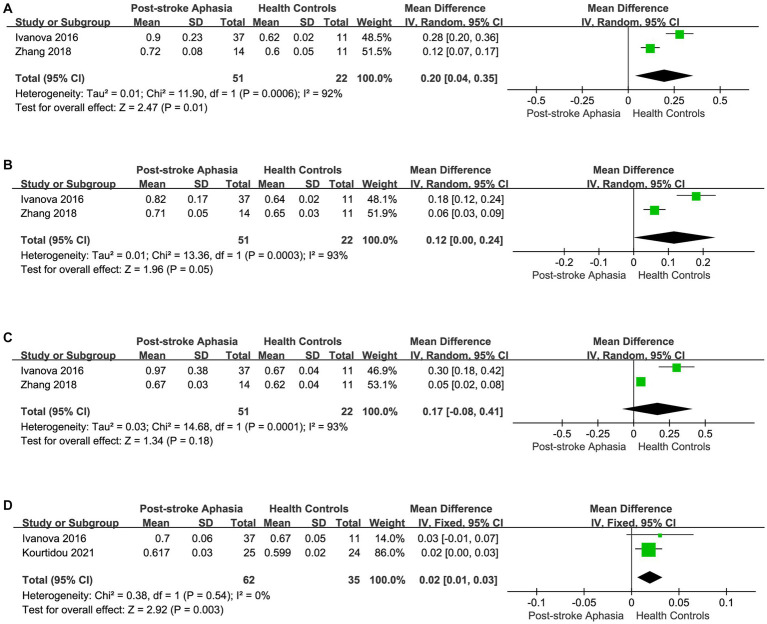
Meta-analysis of the RD in the ventral streams. **(A)** Comparison of RD for IFOF in the LH of PSA and HCs. **(B)** Comparison of RD for ILF in the LH of PSA and HCs. **(C)** Comparison of RD for UF in the LH of PSA and HCs. **(D)** Comparison of RD for UF in the RH of PSA and HCs.

Finally, we performed a the meta-analysis of the RD of IFOF, ILF, and UF in the ventral streams. Two studies reported the RD for IFOF in the LH.The meta-analysis used random-effects to calculate the results, the RD was significantly higher in the PSA group (MD = 0.20; 95% CI = 0.04 to 0.35; *p* = 0.01; *I*^2^ = 92%; 73 participants), but heterogeneity was high (see Figure 6A). Two studies reported the RD for ILF in the LH. The meta-analysis used fixed-effects to calculate the results, AD values were higher in the PSA group than in HCs (MD = 0.12; 95% CI = 0.00 to 0.24; *p* = 0.05; *I*^2^ = 93%; 73 participants), but heterogeneity was higher (see Figure 6B). Two studies reported the RD for UF in the LH. The meta-analysis used random-effects to calculate the results, there was a trend toward higher the RD in the PSA group (MD = 0.17; 95% CI = −0.08 to 0.41; *p* = 0.18; *I*^2^ = 93%; 73 participants), but the differences did not reach statistical significance (see Figure 6C). Two studies reported the RD for UF in the RH. Using a fixed-effects model to calculate the results of meta-analysis, patients with PSA had significantly higher RD than HCs (MD = 0.02; 95% CI = 0.01 to 0.03; *p* = 0.003; *I*^2^ = 0%; 97 participants) (see Figure 6D).

### Broca’s area

**Figure 7:**
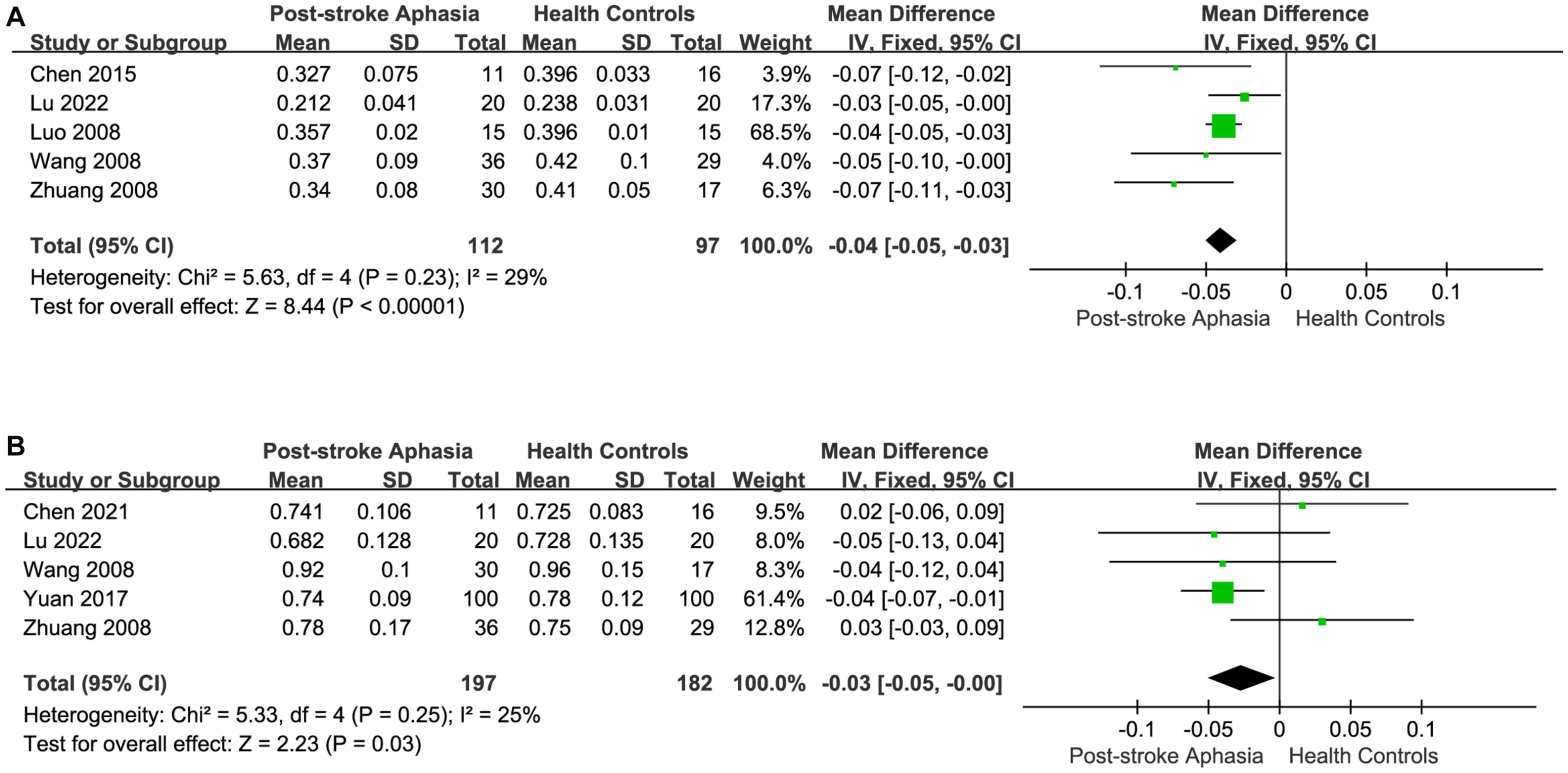
Meta-analysis of the FA and ADC in the Broca’s area of the LH. **(A)** Comparison of FA for Broca’s area in the LH of PSA and HCs. **(B)** Comparison of ADC for Broca’s area in the LH of PSA and HCs.

After correlating the different fiber tracts, we performed a statistical analysis of the FA and ADC in studies with Broca’s area as the ROI. Five studies reported FA values in the Broca area in the LH. Using a fixed-effects model to calculate the results of meta-analysis, the FA was significantly lower in the PSA group than in HCs (MD = −0.04; 95% CI = −0.05 to −0.03; *p* < 0.00001; *I*^2^ = 29%; 209 participants) (see Figure 7A). Five studies reported ADC values in the Broca area in the LH. Using a fixed-effects model for the meta-analysis, the PSA group had significantly lower ADC than HCs (MD = −0.03; 95% CI = −0.05 to −0.00; *p* = 0.03; *I*^2^ = 25%; 95 participants) (see Figure 7B).

### Data analysis methods

**Figure 8:**
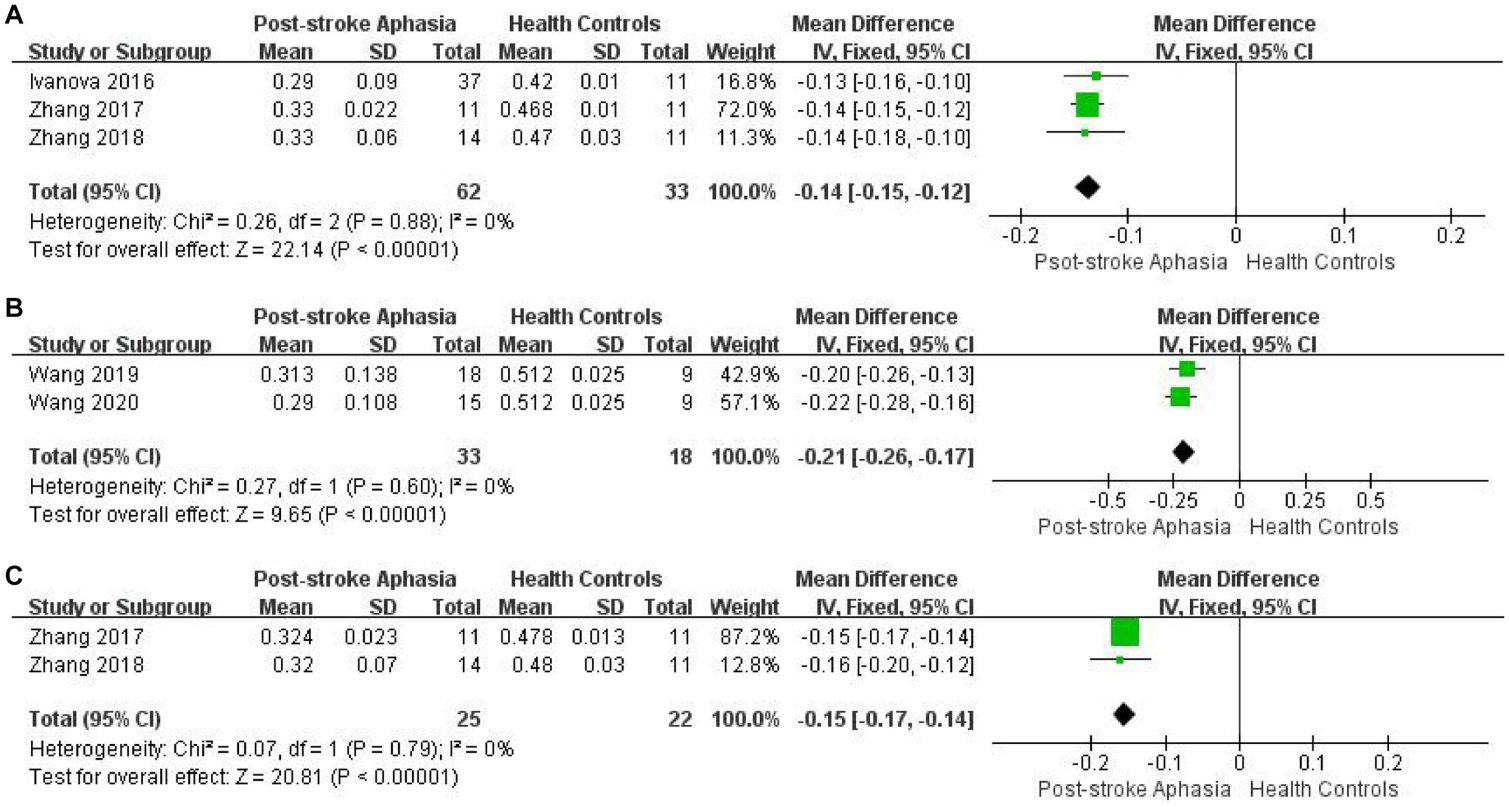
Meta-analysis of FA obtained by different measurement methods. **(A)** Comparison of FA for AF in the LH of PSA and HCs by TBSS. **(B)** Comparison of FA for AF in the LH of PSA and HCs by ROI. **(C)** Comparison of FA for IFOF in the LH of PSA and HCs by TBSS.

In our original design, changes in outcome measures were observed across fiber bundles. However, in some indicators, high heterogeneity was generated. According to the idea of examining clinical heterogeneity, methodological heterogeneity and statistical heterogeneity, we re-analyzed and verified the studies with high heterogeneity. Firstly, we found that the differences in the application and measurement of DTI techniques may be the cause of heterogeneity in FA of AF. As described above, studies of FA in AF in the left hemisphere yielded high heterogeneity (see Figure 2A). In addition, the control group in the study by Koyama and Domen (2016) was described as non-cerebral infarction patients, which may also be one of the reasons. Therefore, we analyzed the FA of AF according to different measurement methods, which significantly reduced the heterogeneity of the studies. Fixed effect model was used for Meta-analysis. In the studies using TBSS method, the FA of PSA patients was significantly lower than that of HCs (MD = −0.14; 95% CI = −0.15 to −0.129; *p* < 0.00001; *I*^2^ = 0%; 95 participants) (see Figure 8A). In the study using ROI analysis method, the FA value of PSA patients was significantly lower than that of HCs (MD = −0.12; 95% CI = −0.26 to −0.17; *p* < 0.00001; *I*^2^ = 0%; 51 participants) (see Figure 8B). We performed the same analysis for FA of IFOF. The lack of information in the study by Ivanova et al. (2016) regarding the time of stroke onset and the type of aphasia among the participants may have contributed to the heterogeneity. Therefore, we performed culling. Fixed effect model was used for meta-analysis. In the studies using TBSS method, the FA value of PSA patients was significantly lower than that of HCs (MD = −0.15; 95%CI = −0.15 to −0.14; *p* < 0.00001; *I*^2^ = 0%; 47 participants) (see Figure 8C). The high heterogeneity in the RD of IFOF, ILF and UF in the ventral stream may also be due to the differences in subjects. In addition, the higher heterogeneity in the study of the RD of AF in the right hemisphere may also be due to the different measurement methods used.

### Systematic review

Of the 8 studies excluded from the meta-analysis, 4 concluded that the FA of AF in the LH was lower in the PSA group than in HCs. One study concluded that the number of impaired voxels in AF correlated with sentence-level comprehension scores. Also, controlling for the confounding effect of the sharpshooter, there was a correlation between the Indicators of DTI in AF in the LH and scales of sentence-level comprehension and word-level comprehension (Xing et al., 2017). One study noted that the mean FA of AF in the LH was positively correlated with auditory sentence comprehension, object naming, rhyme judgments, and homophone judgments, but not significantly correlated with word repetition (Geva et al., 2015). Four studies reported the FA in IFOF, ILF, UF, and SLF in the PSA group, and all 4 studies showed reduced FA in the LH compared to HCs. In one study, WM fiber integrity of SLF and IFOF in ventral and dorsal streams was positively correlated with the aphasia quotient (AQ). involvement of UF integrity correlated mainly with word-level comprehension. Involvement of IFOF integrity correlated with both sentence-level comprehension and word-level comprehension (Xing et al., 2017). According to these findings, word-level comprehension is predominately influenced by the anterior temporal pathway in chronic post-stroke aphasia, whereas the results of sentence-level comprehension is dependent on a broader range of pathways.

At the midpoint of PSA, 2 studies noted a decrease in the FA but an increase in the AD, MD, and RD in the WM fiber tracts of the LH. One of these studies compared PSA group with the Chinese conventional model by TBSS analysis using a general linear model and found a decline in the FA and an elevation in the MD, RD, and AD in PSA group compared with HCs. Moreover, this study pointed out that these changes were mainly concentrated in the left ventral speech pathway and in the SLF of the left dorsal speech pathway. All these results indicate that the microstructure of the LH is impaired. The increase in the RD was associated with demyelination. One study showed an increase in the AD, MD, and RD in the left ventral language pathway, including IFOF, ILF, and UF. Changes in these indicators correlated with word-level comprehension (Xing et al., 2017).

It is noteworthy that in the above study, the main age groups were concentrated in middle-aged and elderly people, but no correlation was found between the annual collar and the DTI index. However, damage to WM fiber structures was observed in all these age groups. We can understand that although some language functions in PSA can be explained by impairments in the dual-stream language pathways of the LH, they cannot be explained by observing fiber tracts in the RH. These findings show that the integrity of WM in the dual-stream language model contributes not only to performance of language but also to overall state cognition. Thus, we believe that aphasia relates to the disruption of multiple connections in the dual-stream, which directly contribute to impairment of language. The damage to the dual streams may be a neural landmark of PSA.

In addition, one study found that patients with PSA exhibited lower volumes of structures of gray matter (Liu, 2021), including areas of the medial inferior frontal lobe (including the olfactory bulb, insula frontalis, and some temporal regions), left superior temporal gyrus, left caudate nucleus, and left middle occipital gyrus, and elevated volumes of gray matter structures in some brain regions, including the right precuneus and right superior temporal gyrus, compared to the HCs by VBM analysis. However, this finding did not correlate with statistical differences in speech assessment.

## Discussion

Based on the meta-analysis, the indicators of DTI of the dorsal and ventral streams in the PSA group showed a trend of higher AD and RD and lower FA compared to the HCs. Among them, it is not difficult to conclude that the change values of major lingual fiber tracts are more significant based on the change of FA, which is strong evidence of fiber integrity and microstructural damage of WM, including AF, SLF, IFOF, ILF, and UF. However, there is a large heterogeneity between the analysis, and different DTI sequence parameters and different anatomical locations may lead to an elevation of the heterogeneity. When we conducted subgroup analysis according to FA for different fiber tracts in the dual-stream model, the heterogeneity of the partial subgroup was significantly reduced. This demonstrates that the source of some of the heterogeneity may arise from differences in anatomical location or fiber alignment. However, the heterogeneity of the FA in AF and IFOF in the LH is still higher than in other WMFs, suggesting that the source of heterogeneity in these two fibers may be more complex and deserves further consideration and exploration (Berthier et al., 2012; Dick and Tremblay, 2012; Almairac et al., 2015; Rollans et al., 2017). It is still significantly correlated with damage to the structure of WM fibers, even though most FA of WM fiber tracts are heterogeneous in the dual-stream language model. Decreasing of the FA can reflect damage to the integrity of the fiber tracts, which also remains consistent with the findings of most studies. Therefore, the changes in WM fiber tracts are one of the essential requirements for the diagnosis of PSA.

It is now generally accepted that Broca’s area of the frontal lobe is connected bidirectionally to Wernicke’s area of the temporal lobe *via* the AF and the adjacent supramarginal gyrus and angular gyrus of the inferior parietal lobule *via* the SLF. Impairment of the integrity of the SLF, including the AF, affects language expression and grammatical processing (Wilson et al., 2011). Among them, it is significant that damage to the AF in the dominant hemisphere is associated with deficits in language repetition, particularly in sentence repetition (Keser et al., 2020). This is an important guide for the localization of clinical aphasia diagnosis. At the same time, the AF and other WM fiber tracts have an important role in conductive aphasia and transcortical aphasia (Davis et al., 1978; Grossman and Irwin, 2018). It is important to gain additional knowledge about the role of neural pathways in the repetition deficits of AF-supported PSA, as it can directly inform prognosis and recovery (Hosomi et al., 2009; Schlaug et al., 2009). Complementary studies combining fMRI and DTI have found activation of superior temporal and prefrontal regions acting through AF during lexical repetition (Kümmerer et al., 2013). Numerous studies have shown that the main changes in indicators of DTI in diseases with nerve fiber tracts injury, including PSA, are the MD, RD, and AD, in addition to the critical FA (Assaf and Pasternak, 2008; Tae et al., 2018). The FA can suggest damage to WM integrity, whereas RD and AD provide a more comprehensive assessment of the microstructure of fiber pathways (Mayo et al., 2018; Podwalski et al., 2021). Subgroup analysis revealed that the FA of the AF in the LH was significantly lower in the PSA group than in HCs, although heterogeneity was higher. The small number of included studies and the heterogeneous distribution of the patient population may be the main factors for the increased heterogeneity. The meta-analysis showed a trend towards higher AD and RD for the AF compared to HCs. However, in the description of AD, the findings of the 2 studies were opposite. One study noted in their study that the AD of the AF in the LH was considered to be reduced compared to HCs (Ivanova et al., 2016), but another study noted in their study that the AD was elevated (Zhang et al., 2018), but both studies noted no statistically significant difference in the AD in the comparison between the PSA group and HCs. This may be due to the difference in the stage of stroke onset in PSA. The elevation of the RD suggests a decrease in vertical motor restriction after demyelination (Klawiter et al., 2011; Winklewski et al., 2018), whereas in the acute phase of stroke axonal injury leads to a decrease in the AD. In the subacute or recovery phase of stroke, the AD appears to decrease with increasing axonal density (Bhasin et al., 2021). This phenomenon also explains the different results of the two studies in our comparison. In addition, the FA of the SLF, another major fiber tract in the dorsal stream, was significantly reduced by subgroup analysis. In the meta-analysis of the RD and AD of the SLF, it was shown that the current study was not sufficient to demonstrate that the elevated RD and AD of the SLF in the PSA group were statistically different when compared with HCs. However, it is noteworthy that changes in FA and RD in the early stages of PSA lead to changes in WM structures, which also indicates the advantage of DTI. This is because, in the early stages, the relationship between structure and function in the brain is less affected by neuronal reorganization (Perez et al., 2021). However, a certain degree of structural and functional change is required for the diagnosis of PSA.

Although the dorsal streams are considered the primary pathway for language processing, as research has progressed, the ventral streams have been recognized as the primary pathway for high levels of language comprehension (López-Barroso and de Diego-Balaguer, 2017). In an earlier study, a cohort of 100 patients with acute PSA used a voxel wise lesion-behavior mapping (VLBM) to identify the brain structures involved in language repetition and comprehension deficits (Rorden et al., 2007) and showed that repetition deficits were significantly correlated with dorsal streams impairment and comprehension deficits were significantly correlated with ventral streams impairment (Kümmerer et al., 2013). In the present study, the meta-analysis showed significantly lower FA of fiber tracts in the ventral streams of the LH in the PSA group compared to HCs, but with high heterogeneity. The small sample size of the included studies may have contributed to this finding. Subgroup analysis showed that the FA of IFOF in the LH was significantly lower in the PSA group than in HCs, but still with high heterogeneity. However, the FA of ILF and UF in the LH were significantly lower in the PSA group than in HCs, with low heterogeneity. We considered that perhaps the elevated heterogeneity of IFOF was due to methodological differences. The reason for this phenomenon may also be due to the narrow and widely distributed nature of the IFOF (Zhang et al., 2018), where methodological differences in its measurement and analysis lead to increased heterogeneity. Despite the heterogeneity, it is possible to characterize the reduced FA of IFOF in the LH of the PSA group. It has been demonstrated in many studies that the FA of IFOF in the LH is positively correlated with reading, naming, and comprehension (Duffau et al., 2005; Harvey et al., 2013; Zhang et al., 2021). Some scholars have divided the IFOF into superficial dorsal and deep ventral (Martino et al., 2010), with the latter forming a visual word-shaped area with the syrinx (Sarubbo et al., 2013). The reason for the positive correlation between IFOF integrity and reading and naming processing was explained from an anatomical perspective. According to the meta-analysis, AD of IFOF in the PSA group in the LH did not differ statistically from AD in HCs, and the increase in the RD was more significant, but the heterogeneity was higher, which was considered to be related to the reasons mentioned previously. In addition, compared with HCs, the FA values in both ILF and UF were significantly lower in LHs, whereas AD was not statistically different, and the RD was elevated in UF compared to HCs. The decreased FA and elevated RD of fiber tracts in the ventral streams suggest that axonal injury and demyelination occurred in these fiber tracts, which affected fiber tracts conduction. Ventral streams’ indirect pathway are predominantly composed of the UF and ILF, parallel to the direct pathway of the IFOF (Axer et al., 2013; Duffau et al., 2013). Currently, it has been suggested that UF assists in the whole process of language processing, but strong evidence and clarification of the main division of UF is lacking (Wise, 2003; Nasios et al., 2019). In addition, some studies found that the correct naming of patients was affected after the removal of UF, suggesting that UF is involved in the naming process, especially in the naming of proprietary names (Papagno et al., 2016). As for the ILF, studies are more precise. Semantic processing is closely related to the structural connections between the ILF with the anterior and posterior inferior temporal lobes. Also, there is a positive correlation between plasticity and semantic improvement of the ILF (Rudrauf et al., 2008; Zhang et al., 2018). Although there is still high heterogeneity in the above results, DTI indicators and changes in fiber structure of the WM are still clearly correlated, and changes in WM fiber structure in the dual-stream language model are one of the necessary conditions for the diagnosis of PSA.

In addition to our analysis comparing dual-stream language models, several included Chinese aphasia studies measured Broca’s area as the ROI. The meta-analysis showed reduced FA and ADC in Broca’s area of the LH in the PSA group. This suggests that WM fibers in Broca’s area are disrupted or even broken. In the inferior frontal gyrus, Broca’s area consists of the perisylvian and deltoid cortices, including Brodmann’s area 44 and 45, and is responsible for language production planning and execution, coordinated with posterior parietal and temporal areas through specific bidirectional fiber tracts (Indefrey and Levelt, 2004; Sarubbo et al., 2020). Notably, in some studies, infarcts were not located in the classic Broca’s area, but aphasia occurred. In patients with motor aphasia, some of the infarct were located outside the classical Broca’s area, away from or close to the language center, but the FA appeared reduced and lower than HCs regardless of whether the infarct area occurred in the language area. Based on this observation, the effect of language may be related to the diffusion of the language area (Schlaug et al., 2009). In a recent study, it was found that long-term speech production impairment (After a stroke, the symptoms last for at least 3 months) was predicted by multiple regression analysis only by the degree of damage to the WM directly above the insula near the anterior AF, with no contribution of lesions in Broca’s area to chronic PSA (Gajardo-Vidal et al., 2021). This reflects the advantage of DTI in the diagnosis of PSA.

The important role of RH in language production continues to be reported. It has been shown that the RH processes information related to language production to varying degrees, including in word comprehension, spelling, categorization, and rhyming, and has demonstrated its critical role. More recently, studies have successively affirmed the role of the RH in listening and comprehension (Gajardo-Vidal et al., 2018), as well as its compensatory role when the dominant hemisphere language network is disrupted (Kourtidou et al., 2021). A comparison of WM fiber tracts in the LH was done, followed by a comparison between PSA and HCs in the RH. In the meta-analysis, it was found that the FA was reduced in the PSA group in the dual streams language model in the RH. However, in the dorsal streams, the FA and AD of the AF and SLF in the RH of the PSA group were not statistically different from those in the HCs after subgroup analysis, with a tendency to decrease. However, the RD of AF was elevated in the RH in the PSA group. An analysis of subgroups in the ventral streams revealed a decrease in the FA of the IFOF in the RH. However, the FA of UF was not statistically significantly different. According to the meta-analysis, a higher RD and AD of UF in the RH were found in PSAs when compared to HCs. The above results suggest that we perhaps received the effect of LH injury in the RH, resulting in damage to the fiber tracts of the dual-stream in the RH as well. However, there were also discrepant results in these results, leading to a partial contradiction between the results. We consider that this may be related to the stage of the condition or the degree of aphasia of the patients included in the study. A previous study found that patients with chronic aphasia showed normalization of activation in the RH in language-related areas and a re-shift of peak activation to the LH. Impairment of the integrity of the WM in the RH is associated with a poor prognosis for aphasia (Saur et al., 2006). The RH has been studied extensively in order to determine its role in PSA recovery, but results have also been contradictory. In the LH, FA values of AF and SLF are correlated with speech repetition, but they are not correlated with speech performance in the right hemisphere. In addition, there was no difference in FA between PSA group and HCs for RH (Geva et al., 2015). One study suggested that the RH in patients with mild to moderate aphasia has the potential to recover during the treatment of PSA by transcranial magnetic stimulation, as detected by PET and fMRI. However, this study did not mention patients with severe aphasia, so the WM microstructure of both hemispheres may be damaged in patients with severe aphasia. This damage resulted in original inferiority of the RH to the LH and thus maladaptation occurred, preventing the recovery of the dominant side (Gainotti, 1993; Thulborn et al., 1999; Martin et al., 2009). Taken together, this evidence suggests that we should remain skeptical when using the DTI technique for the diagnosis of PSA in the dual-stream language model of RH. These contradictory results still require extensive research to explore and explain.

### Limitation

The DTI has been widely used in the study of PSA. There is a good correlation between WM microstructural abnormalities and this test, but its specificity is limited. We chose DTI indexes as FA, AD, RD, and ADC. misinterpretation is easily generated in the interpretation of these DTI data. In general, lower FA with higher RD can characterize the impairment of fiber bundle integrity. This is mainly the result of the restricted diffusion of water molecules and fiber demyelination. However, changes in indicators at different stages of stroke may not necessarily correlate with structural changes and need to be evaluated in combination with additional measurements and supplementary data. Also, in our study, we still lack RD and AD of some of the fiber tracts in the RH. This makes our results less than perfect, and we will continue to collect relevant data at a later stage. Although we assessed the correlation of indicators of DTI with different aspects of speech production, we did not discuss the different types of PSA in a categorical manner. This is because most of the included studies did not perform an analysis of patients with different types of aphasia. Though the number or sample size of our meta-analysis met the criteria of the base, it was still insufficient (Luo et al., 2018). Secondly, in this study, patients with different types of aphasia were included, which implies the to be highly heterogeneous. There is still a lack of research on the characteristics and differences of lesion sites and mechanisms between different types of aphasia, and the relevant data have not been shown in publications. More in-depth or detailed research is needed to explore the differences. This makes it difficult for us to further analyze and study in PSA. In addition, there is a possibility that the current results may be affected by the average age of the patients and the duration after stroke. The current study lacked data on the duration of follow-up for PSA. It is also known that language function can recover to some extent in the early stage of stroke aphasia, mainly through blood flow reperfusion mechanism. However, neural plasticity and functional reorganization are more important in the late stage of speech function recovery. We also wanted to confirm the difference between early and late PSA using DTI, but relevant data were lacking and could not be obtained completely from publications. Only 12 studies described the duration or time of onset of aphasia after stroke, but limited by the quality of the data, in-depth comparison and correlation analysis could not be conducted. It is possible that these reasons contribute to the heterogeneity of the study and the risk of publication bias. We will continue related studies by further including relevant literature at a future time.

## Conclusion

By this meta-analysis and systematic review, we obtained changes in DTI indicators in the WM fiber tracts and the classical Broca’s area of the dual-stream language model in PSA patients. Although there was high heterogeneity among studies, there was a significant correlation with language impairment. It was more pronounced in different fiber tracts and correlated with processes such as language repetition, comprehension, naming, reading, and expression, respectively. In terms of diagnosis of PSA, DTI is an essential tool, but its specificity is very low. In addition, the recovery process may vary depending on the type of PSA. However, the current data only reflect overall changes in language-related brain areas. DTI alone cannot accurately diagnose PSA. It must be combined with clinical presentation, language assessment scales, and testing by other technical means to assess language impairment in PSA effectively.

## Data availability statement

The raw data supporting the conclusions of this article will be made available by the authors, without undue reservation.

## Author contributions

The study design and planning interpretation of the data, and collection of funding were all conducted by WZ and SD. The manuscript was prepared by HJ and JZ. The literature analysis and search was conducted by WZ. The data collection and entry was contributed by QJ and BL. All authors contributed to the article and approved the submitted version.

## Funding

The National Key R&D Program of China (No. 2018YFC1706001), First Teaching Hospital of Tianjin University of Traditional Chinese Medicine, Exploration and Innovation Project (YB202112), Taishan Scholars Program of Shandong Province (No. tsqn201909186), and Natural Science Foundation of Shandong Province (ZR2019MH056) provided financial support for this study.

## Conflict of interest

The authors declare that the research was conducted in the absence of any commercial or financial relationships that could be construed as a potential conflict of interest.

## Publisher’s note

All claims expressed in this article are solely those of the authors and do not necessarily represent those of their affiliated organizations, or those of the publisher, the editors and the reviewers. Any product that may be evaluated in this article, or claim that may be made by its manufacturer, is not guaranteed or endorsed by the publisher.
